# Effects of *Mimosa pudica* L. leaves extract on anxiety, depression and memory

**Published:** 2016

**Authors:** Ganesh Patro, Subrat Kumar Bhattamisra, Bijay Kumar Mohanty

**Affiliations:** 1 *School of Pharmaceutical Education & Research, Berhampur University, Bhanja Bihar, Berhampur-760007, Odisha, India*; 2 *Department of Pharmacology, Roland Institute of Pharmaceutical Sciences, Berhampur-760010, Odisha, India*; 3 *Department of Botany & Biotechnology, Khallikote Autonomous College, Berhampur-760001, Odisha, India*; 4 *Department of Life Sciences, International Medical University, Bukit Jalil-57000, Kuala Lumpur, Malaysia*

**Keywords:** *M. pudica*, *Dopamine*, *Norepinephrine*, *5- Hydroxytryptamine*, *Acetylcholinesterase*, *Caspase-3*

## Abstract

**Objective:**

The present study was carried out to investigate the neuropharmacological activities of ethyl acetate extract of *Mimosa pudica* (EAMP) leaves on anxiety, depression and memory in a mouse model.

**Materials and Methods:**

Anti-anxiety potential of EAMP was evaluated by elevated plus maze (EPM), light-dark box (LDB) and social interaction (SI) tests in mice.Anti-depressant potential of EAMP was evaluated by forced swimming (FST), tail suspension (TST), and open field tests (OFT). The behavioral findings were further corroborated with estimation of neurotransmitters and their metabolites from mouse brain homogenate. Effect on learning and memory was evaluated by EPM, passive avoidance (PA) tests. Further, it was confirmed with assessment of acetylcholinesterase and caspase-3 activity in brain homogenate.

**Results:**

EAMP showed significant anti-anxiety activity by increasing the time spent in open arm of EPM, light box of LDB. Social interaction time was increased significantly (p<0.01) as compared to vehicle control. There was also significant reduction of immobility time in both FST and TST without any changes in locomotor activity in the OFT. Monoamine neurotransmitters (dopamine and norepinephrine) concentrations were increased significantly (p<0.01) after 4 weeks of treatment as compared to stress control and substantiated the anti-depressant activity. Step down latency was increased (p<0.01) in PA test and transfer latency was decreased (p<0.01) in EPM test of EAMP-treated mice. Acetylcholinesterase and caspase-3 activity was significantly (p<0.05) changed in mice treated with EAMP (200 and 400 mg/kg).

**Conclusion:**

The results revealed that EAMP has anti-anxiety, anti-depressant and memory enhancing activities that are mediated through multiple mechanisms.

## Introduction

Anxiety and its related disorders in individuals with or without dementia are the most common human brain disorders. It is associated with an unpleasant state of tension, apprehension, nighttime awakenings and poorer neuropsychological performance. Depression is one of the most prevalent and life-time threatening forms of mental illnesses (Lucian et al., 2015 ▶). It is a common mood disorder which is associated with loss of interest or pleasure, feelings of guilt or low self-worth, disturbed sleep or appetite, and low energy and affects nearly 17% of the world population and imposes a substantial health burden on societies (Nemeroff, 2007 ▶). According to the WHO, it may become the second cause of illness-induced disability by the year 2020. The monoamine hypothesis suggests that the major neurochemical process in depression is alterations in monoaminergic systems. Effective antidepressant treatments normalize the disturbed monoaminergic systems which are assumed to be responsible for the clinical features of depression (Zheng et al., 2013 ▶). Recent studies have highlighted a strong relationship between depression and dementia. An epidemiological survey revealed that dementia or memory loss is a major hidden problem in Indian populations (Shaji et al., 2002 ▶). The rate of dementia increases exponentially with increasing age and this aging process in mammals is associated with a slow decline of sensory and motor performances in the brain. The decline in sensory and motor performance has been attributed to the oxidative damage to lipids, proteins, nucleic acids and imbalance of various neurotransmitter levels due to oxidative stress. Therefore, various antioxidant supplements and flavonoidal components might be beneficial for preserving brain functions and forestalling the age-related deficits (Sahoo et al., 2014 ▶).

*Mimosa pudica* L. (Family Mimosaceae) is locally known as lajwanti or chuimui in Hindi and is native of Central America, Tanzania, South Asia, East Asia and many pacific Islands (Baby et al., 2013 ▶). The roots and leaves of this plant have been commonly used by tribal people for headache, migraine, dysentery, fever, piles, insomnia, epilepsy, etc (Joy et al., 2001 ▶; Merlin and Narsimhan, 2009 ▶). Also this plant was used as bitter, astringent, acrid, cooling vulnerary, febrifuge, alexipharmic, diuretic, emetic and tonic (Vaidyaratanam, 2001 ▶). In traditional healthcare system, it has been used in the treatment of alopecia, diarrhea, constipation, leprosy, dysentery, insomnia, tumor, blood disorders and various urogenital infections (Chatterjee and Prakash, 2000). Various medicinal and biological properties of this plant anti-diabetic, anti-hepatotoxic, antioxidant, anti-asthmatic, aphrodisiac, sedative and wound healing activities were reported (Sivarajan and Balachandran, 2002 ▶). Phytochemical studies revealed the presence of alkaloids, amino acid, flavonoids glycosides, sterols, terpenoids, tannins and fatty acids in this plant (Tamilarasi and Ananthi, 2012 ▶; Hafsa et al., 2012 ▶). However, until today, there were no reports on neuropharmacological effects of this plant. Hence, the present work was designed to evaluate the anti-anxiety, anti-depressant and memory enhancing effects of ethyl acetate extract of *M. pudica* leaves in mice.

## Materials and Methods

**Drugs and chemicals**

Fluoxetine hydrochloride, 5, 5‑dithiobis‑2‑nitrobenzoic acid (DTNB), acetylcholine iodide, acetyl-Asp-Glu-Val-Asp-p‑nitroanilide, sodium dihydrogen phosphate were procured from Hi‑Media, India. Diazepam hydrochloride (Ranbaxy Laboratories, India) and piracetam (Vetranal, Sigma-Aldrich, USA) were procured. All other chemicals used in this study were of analytical grade.

**Plant material and preparation of the extract**

The leaves of *M. pudica* were collected during the month of November from district Ganjam, Odisha, India. The plant material was authenticated by Dr. B. K. Mohanty (Professor), Department of Botany, K. K. Autonomous College, Berhampur, Ganjam, Odisha (Voucher specimen No- G/3094/2012). The collected leaves of *M. pudica* were washed under running tap water, air-dried and crushed to moderately coarse powder. The powdered leaves (approx. 200 g) were defatted using petroleum ether and then, packed in Soxhlet apparatus for extraction with ethyl acetate at room temperature. After 72 hr of complete extraction, the solvent was removed by distillation and the concentrated extract was dried under reduced pressure at 40 ^o^C in rotary evaporator. A thick semisolid brown paste was obtained and stored in desiccators at room temperature. The extraction yeild was found to be 8.41%. 

**Experimental animals**

Adult Swiss albino mice (20-25 g) of either sex were obtained from Ghosh enterprises, Calcutta, India. They were housed at an ambient temperature of 25±1 °C and 45-55% relative humidity, in polypropylene cages with 12 hr/12 hr light/dark cycles. The animals had free access to standard food pellets (Rayan’s biotechnologies Pvt. Ltd, Hyderabad, India) and water, *ad libitum*. All the experimental protocols were conducted based on the permission of Institutional Animal Ethics Committee (IAEC) of Roland Institute of Pharmaceutical Sciences, Berhampur, India for the purpose of control and supervision of experiments on animal (CPCSEA). The IAEC has approved the experimental protocol (Approval no. 80; 23.03.2013) prior to the animal experimentation. We tried to minimize animals suffering and reduce the number of animals used in the experiments.

**Neuropharmacological investigation of ethyl acetate extracts of** ***M. pudica*** **(EAMP** ***)***

**Assessment for anti-anxiety** **activity:** **Animal grouping and drug treatment**

Swiss mice (25-30 g) were selected for this study and divided into five groups of six animals each. Group I served as vehicle control, Group II served as standard and received diazepam (1 mg/kg/p.o.) once daily for seven days, and Groups III, IV and V served as test group and received EAMP 100, 200 and 400 mg/kg/p.o., respectively once daily for seven days. The experiments were conducted 1 hr after the last drug treatment. The following tests were employed for the evaluation of anxiolytic activity and the animals were used only once in the test.

**Elevated plus maze test (EPMT)**

The plus maze apparatus consisted of two open arms, measuring 16 × 5 cm, and two closed arms, measuring 16 × 5 × 12 cm, connected to a central platform (5 × 5 cm). The maze was elevated to a height of 25 cm above the floor. Each mouse was placed individually at the center of elevated plus maze with its head facing toward an open arm and observed for 5 min to record the number of entries into open arm and closed arm and the time spent in each arm (Kulkarni, 1999; Bhattmisra et al., 2007 ▶). In EPM test, the percentage of time spent on the open arms was determined as follows: % time = 100 × seconds spent on open arms/total seconds (which was 300 sec equal to 5 min observation time).

**Light-dark box test (LDBT)**

The apparatus consisted of a rectangular box (45 × 27 × 27 cm), partitioned into two compartments connected by a 7.5 × 7.5 cm opening in the wall between compartments. Each mouse was placed in the center of the light compartment and the time spent in open (white/light) compartment during 5 min observation was recorded (Crawley and Goodwin, 1980 ▶; File, 1996 ▶). The percentage of time spent in the light compartment was determined as follows: % = 100 × seconds spent in light compartment/ total seconds (which was 300 sec equal to 5 min observation time).

**Social interaction test (SIT)**

The social interaction arena was an open topped box (22 × 15 × 12 cm). Mice were isolated for 1 hr before the test. After introduction to the test arena, mice were observed for cumulative time spent in genital investigation, sniffing a partner, following, grooming, kicking, biting, wrestling, climbing over and under, neck licking and boxing (File, 1996 ▶).

**Assessment for anti-depressant** **activity**

**Animal grouping and drug treatment**

Swiss mice (25-30 g) were divided into six groups of six animals each. Group I served as vehicle control, Groups II served as stress control and was exposed to stress induction only, Group III served as standard and received fluoxetine (5 mg/kg/p.o) once daily for 4 weeks with stress induction and Groups IV, V and VI served as test groups and received EAMP 100, 200 and 400 mg/kg/p.o., respectively once daily for 4 weeks with stress induction. 

 The stress was induced by following procedures i.e. tilting of cage (45° with 23hr: 1hr); empty water bottles (23hr: 1hr); food or water deprivation (23hr: 1hr); cold water swimming (4 °C for 5 min); continuous overnight illumination (24 hr); intermittent illumination (light on and off every 2 hr); soiled cage (100 ml of water spilled onto the bedding (23 hr). Stressor methods were employed individually each day for 4 weeks. The following anti-depressant models were conducted 1hr after the last drug treatment.

**Forced swimming test (FST)**

Mice were individually forced to swim in an open cylindrical container (diameter 10 cm and height 25 cm), with a water depth of 19 cm at 25±1^o^C; the total amount of time each animal remained immobile during a 6-min session was recorded (in seconds) as immobility time (Machado et al., 2009 ▶). Each mouse was considered immobile when it ceased struggling and remained floating motionless in the water, making only movements necessary to keep its head above water. A decrease in the duration of immobility is indicative of an anti-depressant like effect (Porsolt et al., 1977 ▶; Bhattmisra et al., 2008 ▶). 

**Tail suspension test (TST)**

The mice were individually suspended by the tail with a clamp (10 mm from the tail tip in a box (250 × 250 × 300 mm) with the head 50 mm from the bottom. Test was carried out in a dark room with minimal background noise. Each mouse was suspended for a total of 6 min, and the duration of immobility was recorded during the final 4 min interval of the test. Mice were considered immobile only when they hung passively and completely motionless. This test is a reliable and rapid screening method for anti-depressants including those affecting the serotonergic system (Steru et al., 1985 ▶; Bhattmisra et al., 2008 ▶). 

**Open field test (OFT)**

The mice were individually housed in a rectangular container made of dark polyethylene (40 × 40 × 25 cm) in a dim-lighted room equipped with a video camera above the center of the floor and locomotor activity was measured (Kim et al., 2007 ▶). The animals were allowed to adapt for 1 hr in the container, and the distance they travelled was recorded during the last 10 min of a total 20 min test. The locomotor activity was measured in centimeters. The floor surface of each chamber was thoroughly cleaned with 70% ethanol between tests. 

**Determination of neurotransmitters and their metabolites from brain homogenate of mice**

**Sample preparation**

Pretreated mice with stress for 4 weeks were used for the determination of neurotransmitters and their metabolites concentration in brain. The animals were sacrificed and the whole brain was carefully removed. The brain tissues were immediately placed on ice, transported in liquid nitrogen and frozen at - 80 ^o^C until biochemical analysis. The cerebral tissues were homogenized in ice cold methanol after being precisely weighed. One milliliter of homogenate was pipette into 1.5 ml conical plastic centrifuge tube and centrifuged at 14,000 rpm for 20 min at 4 ^o^C. Then, the supernatant was concentrated by nitrogen evaporator. The residue was then reconstituted with 300 µl deionized water and vortex-mixed for 10 sec. Then, 300 ml chloroform-isopropanol (100:30, v/v) was added. After, vortex-mixing for 2 min, the mixture was centrifuged at 3,000 rpm for another 5 min at 4 ^o^C. The upper aqueous layer was filtered through a 0.45 mm filter prior to use (Zheng et al., 2013 ▶; Haixia et al., 2009 ▶). 

 The UPLC-MS/MS system used in this analysis consisted of an acuity UPLC (Waters, Milford, MA) coupled to a quadrupole mass spectrometer. The column used was a pentafluorophenyl column (2.1 × 150 mm, 1.9 µm). The eluents used were aqueous 0.1% formic acid, acetonitrile and 0.1% formic acid. The flow rate was 0.3 ml/min, and the column oven temperature was kept at 30 ^o^C. The injection volume was 15 µl. The flow from the UPLC was directed to waste for the first minutes of the gradient to avoid contamination of the ion source by the salts of the Ringer’s solution or the cerebrospinal fluid. A quadrupole mass spectrometer with an electrospray ion source was used in the quantitative analyses of the brain samples. Nitrogen was used as the nebulizer (40 psi), curtain (12 l/min, 350 ^o^C), and collision gas. The fragmentor voltages and collision energies were optimized for each compound and the software (MassLynx V 4.1 software) was used for quantitative and qualitative data analysis. The compound-specific mass spectrometric parameters had been optimized earlier (Uutela et al., 2009 ▶). The positive and negative ion ESI mass spectra showed abundant [M+H]^+^ and [M-H]^-^ ions, which were chosen for the precursor ions. The identification of the neurotransmitters and their metabolites was based on the comparison of the retention times and relative abundances of each analyte between the reference standards diluted in Ringer’s solution and the authentic samples. The samples were analyzed in two runs using the same chromatographic gradient but monitoring different transitions in order to maximize sensitivity and selectivity. 

**Assessment of memory activity**

**Animal grouping**

Healthy male Swiss mice were selected and divided into five groups of six mice each. Group I served as vehicle control, Group II was treated with standard drug (Piracetam 400 mg/kg/i.p.) and Groups III, IV, and V were administered with EAMP 100, 200 and 400 mg/kg/p.o., respectively which were administered daily for 15 days. The following tests were employed for the evaluation of memory activity, 1 hr after the administration of the extract and the animals were used once for each test.

**Elevated plus maze (EPM) test**

The apparatus consisted of two open arms (16 × 5 cm) and two closed arms (16 × 5 × 12 cm) that extended from a common central platform (5 × 5 cm). The entire maze was elevated to a height of 25 cm above the floor level. After the 15th day of drug treatment, each mouse was placed at the end of an open arm, facing away from the central platform. Transfer latency (TL) was recorded for each group and compared to control group. TL was the time (in seconds) spent by the animals to move from the open arm into any one of the covered arms with all its four legs. The reduction in TL value of retention indicated improvement in memory (Bhattamisra et al., 2012 ▶). 

**Passive avoidance (PA) test**

The apparatus consists of a box (27 × 27 × 27 cm) having three walls of wood and one walls of plexiglass, featuring a grid floor (made up of 3 mm stainless steel rods set 8 mm apart), with a wooden platform (10 × 7 × 1.7 cm) in the center of the grid floor. The box was illuminated with a 15 W bulb during the experimental period. Electric shock (20 V, A.C.) was delivered to the grid floor. Training (i.e. 15th day of drug treatment) was carried out in two similar sessions. Each mouse was placed on the center of the grid floor. When the mouse stepped down with all paws on grid floor, shocks were delivered for 15 sec and the step‑down latency (SDL) was measured. SDL was recorded after 15th day of drug treatment for each treated group and compared to control group as passive avoidance behavior for each trial (Bhattamisra et al., 2012 ▶). 

**Collection of brain sample**

After the 15th day of administration, the animals of all groups were sacrificed by cervical decapitation under light anesthesia, 90 min after the last dose. The whole brain (without the cerebellum) was removed carefully from the skull and transferred to a glass homogenizer. The fresh whole brain was homogenized in an ice bath with 10 volumes of normal saline injection. The homogenate was centrifuged at 3,000 rpm for 10 min at 4 ^o^C and the pellets were re-extracted with an equal volume of 30 mM Na_2_HPO_4_, pH 7.6, containing 1% Triton X‑100 and the suspensions were centrifuged at 10,000 rpm for 2 hr at 4 ^o^C. The resultant supernatant was used for the estimation of brain cholinesterase activity, and the pellet for caspase-3 activity. For estimation of cholinesterase and caspase‑3 activity, acetylthiocholine iodide and acetyl-Asp-Glu-Val-Asp-p‑nitroanilide (Ac-DEVD‑pNA) were taken as substrates, respectively by colorimeter. The AChE activity was expressed as mmol/min/g of tissue protein whereas caspase‑3 was expressed as nmol/h/mg of protein (Sahoo et al., 2014 ▶). 

**Statistical analysis**

The values were expressed as Mean ± SEM. Statistical analysis was done by one-way ANOVA followed by Dunnett’s multiple comparison test vs. control. p<0.05 and p<0.01 were considered statistically significant.

## Results

**Effect of EAMP on anxiolytic models of EPMT, LDBT and SIT in mice**

**Figure 1:**
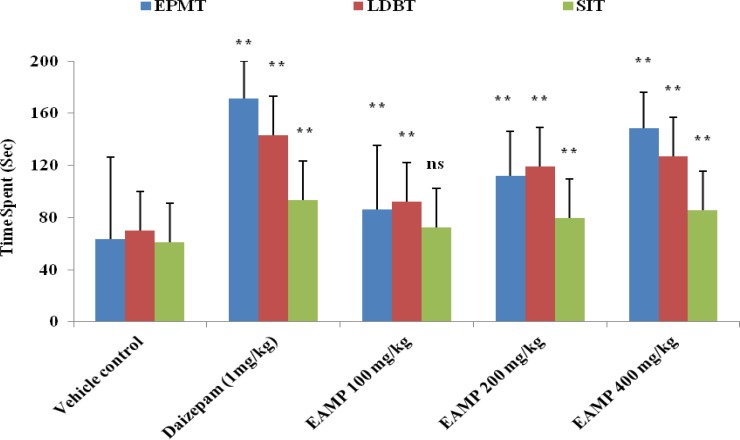
Effect of EAMP on anxiety models of EPMT, LDBT and SIT in mice. EAMP (100, 200 and 400 mg/kg, p.o.) and diazepam (1 mg/kg, p.o.) were administered once daily for 7 days. Each value represented Mean ± SEM of six mice. The data were analyzed using one way ANOVA followed by Dunnett’s test. ** p< 0.01 shows significant difference as compared to vehicle control group

In EPMT, all the doses of EAMP significantly (p< 0.01) increased the time spent in the open arm as compared to vehicle control. But, EAMP 200 and 400 mg/kg had no significant effect on number of entries into the open arm where as EAMP 400 mg/kg was able to cause significant (p<0.01) change as compared to vehicle control. Similarly in LDBT, significant (p<0.01) increase in the time spent in light compartment was seen with all doses of EAMP as compared to vehicle control. In SIT, EAMP 200 and 400 mg/kg significantly increased the time spent in social interaction as compared to vehicle control. No significant effects were observed at 100 mg/kg of the plant extract of EAMP. All the doses of EAMP showed dose-dependent anxiolytic effect against EPMT, LDBT and SIT as shown in Figure 1.

**Effect of EAMP on FST, TST, OFT and brain transmitter in stress-induced mice**

**Figure 2:**
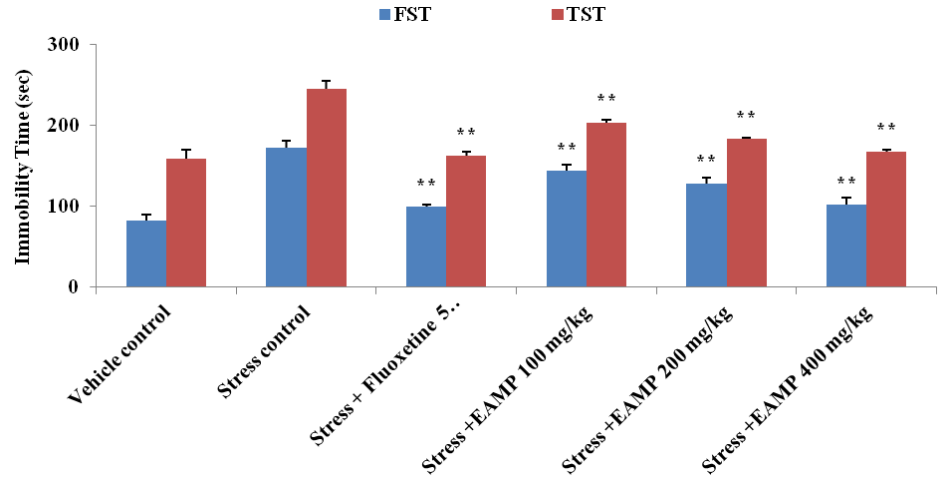
Effect of EAMP on immobility time of forced swimming test and tail suspension test in mice. EAMP (100, 200 and 400 mg/kg, p.o.) and fluoxetine (5 mg/kg/p.o) were administered once daily for 4 weeks following stress induction. Each value represented Mean ± SEM of six mice. ** p <0.01 shows significant difference as compared to stress control by one way ANOVA followed by Dunnett’s test

Immobility time in FST and TST was significantly (p<0.05) reduced after treatment with EAMP (100, 200 and 400 mg/kg) and fluoxetine (5 mg/kg) as compared to stress control group (Figure 2). In open field test, the movement distance and movement time of EAMP were increased significantly (p<0.05) as compared to stress control group. The results are mentioned in Table 1.

**Figure 3:**
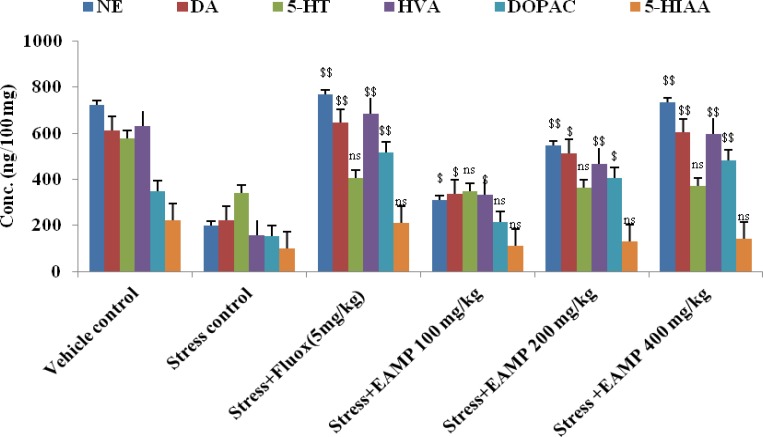
Effect of EAMP on NE, DA, 5-HT and their metabolites concentration (ng/100 mg) in mice brain. NE: Noradrenaline, DA: Dopamine, 5-HT: Serotonin, HVA: Homovanilic acid, DOPAC: Dopa carboxylase and 5-HIAA: 5- hydroxyl indole acetic acid. EAMP (100, 200 and 400mg/kg, p.o.) and fluoxetine (5 mg/kg/p.o) were administered once daily for 4 weeks following stress induction. Each value representss Mean ± SEM of six mice. $$p<0.01 and $p<0.05 show significant difference as compared to the stress control. ns: Not significant

The content of catecholamines from the brain homogenate after 4 weeks treatment is shown in Figure 3. The levels of DA, NE, 5-HT and their metabolites in mice brains with stress-induced depression group were reduced significantly (p**<**0.01, p**<**0.05) as compared to the vehicle control group. After 4 weeks of treatment with fluoxetine (5 mg/kg/p.o), DA, NE, 5-HT and their metabolite levels were markedly (p**<**0.01, p**<**0.05) increased as compared to the stress-induced control group. EAMP (400 mg/kg) significantly (p**<**0.01) increased NE and DA and its metabolites (DOPAC, HVA levels), but 5-HT and its metabolites (5-HIAA) levels were unchanged after 4 weeks of treatment.

**Table 1:** Effect of EAMP on the movement distance and movement time in open field test in mice

**Treatment**	**Movement distance (cm)**	**Movement time (sec)**
**Vehicle control**	2133.14±12.32	139.09± 7.86
**Stress control**	900.53±14.65	56.18±4.32
**Stress + Fluoxetine (5 mg/kg)**	2008.45±21.42**	127.72±8.99**
**Stress +EAMP (100 mg/kg)**	1653.84±18.52**	98.21±6.46**
**Stress +EAMP** ** ( 200 mg/kg)**	1715.92±13.49**	111.33±4.23**
**Stress +EAMP (400 mg/kg)**	1999.71±19.23**	125.54±8.41**

EAMP (100, 200 and 400mg/kg, p.o.) and fluoxetine (5 mg/kg/p.o) were administered once daily for 4 weeks following stress induction. Each value represented Mean ± SEM of six mice. The data was analyzed using one way ANOVA followed by Dunnett’s test.

** p < 0.01 shows significant difference as compared to stress control group.

**Effect of EAMP on TL, SDL and brain enzymes activity in mice**

**Figure 4:**
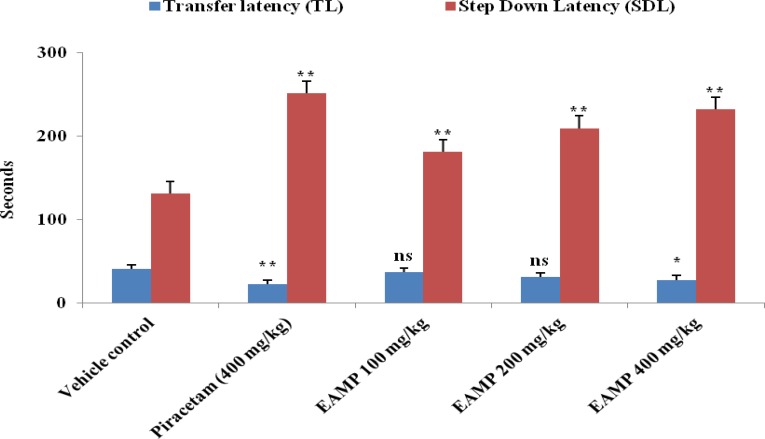
Effect of EAMP on the transfer latency and step down latency in mice. EAMP (100, 200 and 400mg/kg, p.o.) and piracetam (400 mg/kg/i.p.) were administered once daily for 15 days. Each value represents Mean ± SEM of six mice. The data was analyzed using one way ANOVA followed by Dunnett’s test. ** p<0.01 and * p<0.05 show significant difference as compared to vehicle control group. ns: Not significant

 The EAMP 400 mg/kg significantly (p**<** 0.05) decreased transfer latency after 15^th^ day of treatment in mice whereas, EAMP 100 and 200 mg/kg did not exhibit significant effect on transfer latency (TL) as compared to vehicle control (Figure 4). There is a significant (p**<** 0.01) decrease in step down latency (SDL) at all the doses of EAMP along with standard group and all the doses of EAMP showed a dose-dependent memory enhancing effect as compared to control (Figure 4). EAMP (200 and 400 mg/kg) and piracetam (400 mg/kg) as the standard, showed remarkable reduction in brain cholinesterase activity in mice, as compared to control group as shown in Table 2. But, EAMP (400 mg/kg, p.o.) showed a more marked (p<0.01) reduction of brain cholinesterase activity in mice after 15th day of treatment. Caspase-3 activity in brain homogenate was significantly (p<0.05) augmented in EAMP 200 mg/kg and p< 0.01 in EAMP 400 mg/kg and piracetam 400 mg/kg treatment. The result is illustrated in Table 2. 

**Table 2:** Effect of EAMP on cholinesterase and caspase‑3 level in brain homogenate of mice

**Treatment**	**Cholinesterase (mmol/min/g)**	**Caspase** **‑** **3 (nmol/h/mg)**
**Control**	29.13±2.17	43.21±2.47
**Piracetam (400mg/kg)**	17.91± 1.76**	64.19±3.17**
**EAMP (100mg/kg)**	25.28±1.52ns	48.83±2.59 ns
**EAMP (200mg/kg)**	21.72±2.81*	55.45±1.45*
**EAMP (400mg/kg)**	19.43±1.18**	61.28±3.11**

EAMP (100, 200 and 400mg/kg, p.o.) and piracetam (400 mg/kg/i.p.) were administered once daily for 15 days. Each value representss Mean ± SEM of six mice. The data was analyzed using one way ANOVA followed by Dunnett’s test.

** p<0.01 and

* p<0.05 show significant difference as compared to control group. ns: Not significant.

## Discussion

Anxiety disorders are cognitive dysfunction associated psychopathologies that are almost inevitably encountered in many medical and surgical conditions. Currently available psychoactive drugs, mainly anxiolytics and anti-depressants do not often properly meet the therapeutic demands of patients suffering comorbid psychiatric conditions, and the drawbacks of such drugs in terms of unwanted side effects, incredible benefits and moderate costs (Gireesh et al., 2013 ▶). So, herbal plants are good sources to find new remedies for these disorders. In the search for an alternative, more specific and perhaps cost effective therapy, research has been conducted to investigate natural anxiolytic drugs as well as new anti-depressant principles. The elevated plus maze is considered to be an etiologically valid animal model of anxiety because it uses natural stimuli viz. fear of a novel open space and fear of balancing on a relatively narrow, raised platform that can induce anxiety in humans (Grundmann et al., 2007 ▶). The ratio of open/closed area entries and the time spent reflect a specific effect on anxiety. In the present study, oral administration of EAMP (100, 200 and 400 mg/kg) exhibited an anxiolytic-like effect in mice, since it increased the number of entries and the time spent on open arms and decreased the time spent in closed arms in the EPM test. In agreement with previously published reports, diazepam increased the percentage of time spent on open arms and the number of entries into the open arms (Tokumo et al., 2006 ▶). The social interaction test and light/dark box of anxiety were developed to provide an ethologically-based test which are sensitive to both anxiolytic and anxiogenic effects. Generally speaking, an increase in social interaction is indicative of an anxiolytic effect, whereas a specific decrease in social interaction indicates an anxiogenic effect. This test provided a new approach to evaluate the neurobiological mechanisms underlying anxiety disorders (kumar et al., 2012 ▶). Light/dark box test is based on the innate aversion of rodents to brightly illuminated areas and on the spontaneous exploratory behavior of rodents in response to mild stressors (i.e. a novel environment and light). 

It has been reported that simple measurement of the time spent in the light area, but not the number of transfers, is the most consistent and useful parameter for assessing an anxiolytic action (Wei et al., 2007 ▶). Mice treated with EAMP (100, 200 and 400 mg/kg) showed increase in the time spent in the light compartment and no changes in the numbers of shuttle crossings, confirming the effect on the main anxiolytic parameter. The observed anxiolytic effect of EAMP may be due to an agonistic effect on GABA/benzodiazepine receptor complex, an antagonistic effect on 5-HT_1B_ receptors, or an agonistic activity on 5-HT_1A_ receptors (Thippeswamy et al., 2011 ▶). EAMP possesses anxiolytic activity as similar to diazepam that acts via the GABA receptor complex, as flavonoids and diazepam are structurally similar. Flavonoids and alkaloids in many plant species used as folk medicine, exert anxiolytic activity (Elisabetsky and Costa-Campos, 2006 ▶; Poonam and Shradha, 2011 ▶). So, anxiolytic activity of EAMP may be due to the presence of flavonoids and alkaloids.

Mild stress is generally thought to be the most promising and valuable rodent model to study depression, as it mimics several human depressive symptoms and is more suitable for studying the neurobiological basis of depression (Willner et al., 1992 ▶). Therefore, in the present study, we investigated the antidepressant effects by inducing mild stress for 4 weeks in mice. Behavioral study plays an important role in the evaluation and development of anti-depressant drugs. The tail suspension test and forced swimming test are widely used to detect and characterize the efficacy of new anti-depressant drugs along with their neurobiological mechanisms (Bourin et al., 2005 ▶). These animal models were based on the despair or helplessness behavior in some inescapable and confined space in animals and are sensitive to various anti-depressant drugs. The present result confirmed that administration of EAMP (100, 200 and 400 mg/kg) had a specific anti-depressant-like effect in both FST and TST in mice by significantly reducing the immobility time as compared to stress control and fluoxetine-treated group. Moreover, the anti-immobility effect produced by EAMP shared some pharmacological mechanisms with established anti-depressant drugs in this investigation and showed dose-dependent anti-depressant effect. Similar outcome were obtained in TST as EAMP (100, 200 and 400 mg/kg) significantly reduced the immobility time. These results indicate that EAMP has a dose-dependent anti-depressant like effect that is comparable to established anti-depressant drugs. Hence, the anti-depressant action of EAMP is possibly mediated through one of the mechanisms of anti-depressant agents that are effective in TST. Again, FST and TST increase the cortisol level of mice by altering hyperactivity (shalam et al., 2007 ▶). EAMP showed anti-depressant effect which was probably due to reduction of the corticosterone concentration in mice exposed to FST and TST. In fact, hyperkinesia also causes false positive effect in FST and TST by shortening the immobility time in both tests. Therefore, OFT was used to exclude these false effects that could be associated with hyperkinesia. The main difference between antidepressants and psycho-stimulants is that antidepressants do not increase general motor activity (Farah et al., 2011 ▶). In our study, repeated administration of EAMP did not increase locomotor activity at doses that produced an antidepressant-like effect, indicating that the specific actions of this extract on the behavioral model are predictive of anti-depressant activity. In addition, the antidepressant effect of EAMP was not influenced by changes in locomotor activity (i.e. by hypoactivity).

The neurochemical mechanism of depression is due to the impairment of monoaminergic functions (i.e. the decrease of serotonin, noradrenaline and dopamine levels). Anti-depressant drugs increase the availability of these monoamines at the synapse, which may promote longer term adaptive changes by modulating monoaminergic functions and initiating neurogenesis (Zheng et al., 2013 ▶). Using UPLC/MS analysis, the monoamine neurotransmitter system is decreased in stress-induced control group which may mediate the behavioral abnormalities (e.g. hypoactivity, hyponeophagia and anhedonia). The present study showed that treatment with EAMP (100, 200 and 400 mg/kg) reverses stress-induced decrease in NE and DA and its metabolites DOPAC and HVA levels significantly (p<0.05, p<0.01), but 5-HT and its metabolites 5-HIAA levels remained unchanged in comparison with the stress-induced control group. EAMP (400 mg/kg) showed similar antidepressant potential compared to fluoxetine (5 mg/kg) in behavioral tests. 5-HT system plays a major role in depression (Jans et al., 2007 ▶) but, our results demonstrated that there is no significant difference in the levels of 5-HT and 5-HIAA between the stress control and the drug-treated group. Here, the impairment of the serotonergic system in stress control may occur at other sites, but the concentration of 5-HT was not changed. In addition, brain-derived neurotrophic factor (BDNF) has potent neurotrophic factor of neuronal populations (noradrenergic, serotonergic, dopaminergic, cholinergic, and GABAergic neurons). BDNF is an important modulator of progenitor cell proliferation, differentiation and survival (Schmidt and Duman, 2007 ▶). On the other hand, increased monoamine neurotransmission can also induce neuronal BDNF expression. Thus, it can be hypothesized that EAMP may exert anti-depressant like activity by regulating the interaction between monoamine system and BDNF, thereby, modulating the neuronal survival, neuroplasticity and neurogenesis. 

The relationship between dopaminergic system and depression was confirmed by the fact that anti-depressants act on the dopaminergic system. Common symptoms of depression such as anhedonia, dysphoria, and avolition may be caused by a functional deficit of dopaminergic transmission. Furthermore, reports suggest that severity of depression is inversely correlated with central nervous system dopamine metabolite levels (Zheng et al., 2013 ▶). These results indicate that an activation of dopamine D_1_ and D_2_ receptors are likely implicated in the anti-depressant like effect of EAMP in the tail suspension test. It is possible that an activation of the dopaminergic system elicited by EAMP may be a mechanism underlying its anti-depressant like effect that may be beneficial for the treatment of depression associated with anhedonia.

The beneficial effect of EAMP on memory performance in mice was assessed using elevated plus maze and passive avoidance task. In the present study, EAMP administration increased step down latency in passive avoidance task and decreased transfer latency in elevated plus maze test as compared to vehicle control. The observed behavioral results using elevated plus maze test and passive avoidance test showed that administration of EAMP 400 mg/kg for 15 days caused significantly higher results during acquisition and retention of memory as compared to control group. It does clearly indicate that oral administration of EAMP has enhanced learning and retrieval ability of previously acquired information providing additional support to the earlier reports. Aging is specially characterized by an impairment of cognitive function, including learning and memory. The regulation of memory function depends upon the levels of neurotransmitter such as acetylcholine (ACh), choline acetyltransferase (ChAT) and acetyl cholinesterase (AChE) which are critical components of Alzheimer’s disease (Terry and Buccafusco, 2003). In addition, some studies have shown an indirect relationship between age-related changes in memory function and the cholinergic system after either brain lesions and/or administration of anti-cholinergic drugs in both human and animals (Darreh-Shori et al., 2006 ▶). AChE is an important regulatory enzyme that metabolizes acetylcholine to choline and acetyl-CoA, at brain cholinergic synapses as well as the neuromuscular junction (Lane et al., 2006 ▶). It is expected that decreased AChE activity may enhance cholinergic activity by raising ACh level within the CNS thereby improving cognitive functions in rats (Smith et al., 1996 ▶) and monkeys (Rupniak et al., 1997 ▶). Blockade of AChE results in an increased level of ACh at synapse and augmentation of cholinergic neurotransmission. Many cholinergic agonists, reversible AChE inhibitors such as physostigmine, tacrine, donepezil and rivastigmine have been used as cognitive enhancers in the treatment of Alzheimer’s disease and other dementia disorders (Lane et al., 2006 ▶). Pre-treatment with EAMP (200 and 400 mg/kg) significantly attenuated AChE activity resulting in an increase in the basal level of acetylcholine in mice. Therefore, the present investigation indicates that EAMP might be responsible for maintaining learning and memory functions.

Recently, Dash et al. (2000) ▶ reported that caspase-3 plays an essential role in long-term memory. The differential expression of caspase family proteins during development and aging as well as differential subcellular localization in adult rats brain indicates that caspases may contribute to regulation of synaptic plasticity (Shimohama et al., 2001a ▶,b ▶). It has been demonstrated that treatment of rat hippocampal slices with a caspase-3 inhibitor led to decreases in the magnitude of long-term potentiation (Gulyaeva et al., 2003 ▶). In addition, administration of a caspase-3 inhibitor exhibited impairment of learning and memory processes both in water maze test and in acquisition of a conditioned active avoidance reflex (Dash et al., 2000 ▶; Stepanichev et al., 2005 ▶). Moreover, caspases maintaining normal long-term neuroplasticity through the possible involvement of calpastatin (endogenous calpain inhibitor), cytoskeletal proteins actin and fodrin (-spectrin), and components of signal transduction such as inositol-3-phosphane receptor, protein kinase C, Ca^2+^-calmoduline kinases, focal adhesion kinase, Fyn (Src) tyrosine kinase, protein phosphatase 2A, and phospholipase A_2_ (Gulyaeva, 2003 ▶; Gulyaeva et al., 2003 ▶). The caspase-3 level is mainly increased due to the presence of carotenoids and flavonoids (Papandereou et al., 2011 ▶). In our observation, EAMP 200 and 400 mg/kg remarkably increased caspase-3 levels in mice brain, which may be due to the presence of these phytoconstituents. Dietary supplementation with polyphenols, carotenoids, flavonoids and fatty acids exerts beneficial effects not only through scavenging of free radicals, but also by modulating signal transduction, gene expression, and restoring optimal neuronal communication (Farooqui and Farooqui, 2009 ▶). A combination of anti-inflammatory, antioxidant and neuroprotective activity and the presence of flavonoids and phenolic compounds could all lead to net improvement of memory activity (Sahoo et al., 2014 ▶). 

The present investigation not only confirmed the beneficial effect of ethyl acetate extract of *M. pudica*, but also validated its effects on anxiety, dementia and depression-like symptoms. Moreover, our results demonstrated that ethyl acetate extract of *M. pudica* exerts potent anti-depressant like effects in behaviors involve the normalization of neurochemical abnormalities in the monoamine neurotransmitter system. Behavioral effects on learning and memory were augmented by EAMP and it was established through attenuation of acetylcholinesterase activity and augmentation of aspase-3 activity. However, further studies are required for its putative role in neuroprotection and behavioral improvement.
